# Supplementary material for: Single nuclei transcriptomics in human and non-human primate striatum in opioid use disorder
Source: Nat Commun. 2024 Jan 31;15:878. doi: 10.1038/s41467-024-45165-7 (PMC10831093; doi:10.1038/s41467-024-45165-7)
Supplement: Supplementary file 1 — Supplementary Information [file 41467_2024_45165_MOESM1_ESM.docx]

### Supplementary Figures


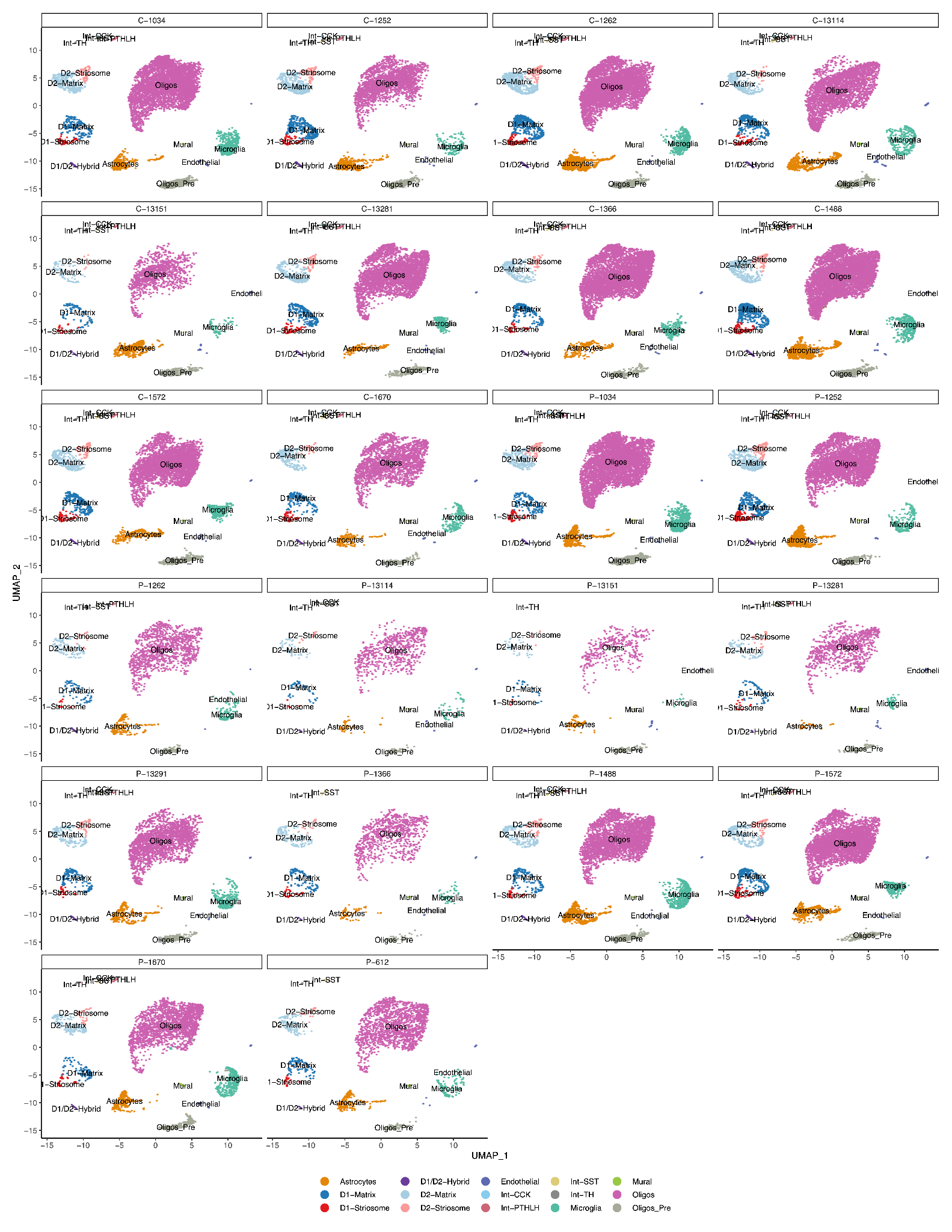


#### Supplementary Figure 1. Low dimensionality projection of striatal cell types after QC filtering and annotation.

#### Cell clusters are based on marker genes, with each cell cluster represented by a different color. Cell clusters are plotted for each subject (number) and tissue type (C: caudate; P: putamen). Source data are reported in “Figure1_source_data.xlsx”.

#### Supplementary Figure 2. Marker genes for dopamine receptor subtype medium spiny neuron subpopulations.
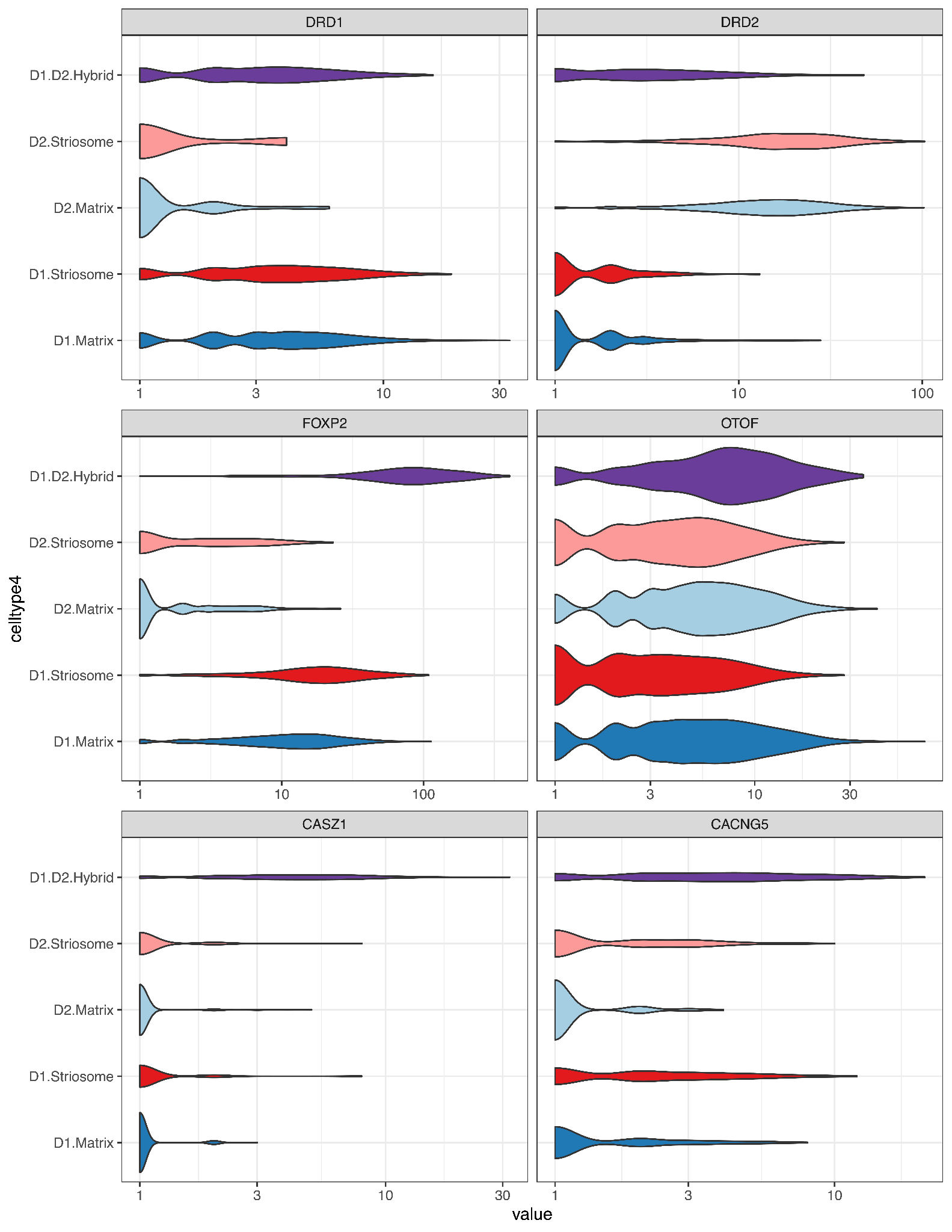


#### Violin plots showing normalized counts for marker genes by major dopamine receptor medium spiny neuron subtype. The mouse orthologs of OTOF, CASZ1, and CACNG5 are marker genes for a previously described striatal cell type differently termed “eccentric spiny projection neuron”, D1-hybrid, or D1-*Pcdh8* neurons. Exact numbers of each cell type are reported in Source data Figure1_source_data.xlsx.

####
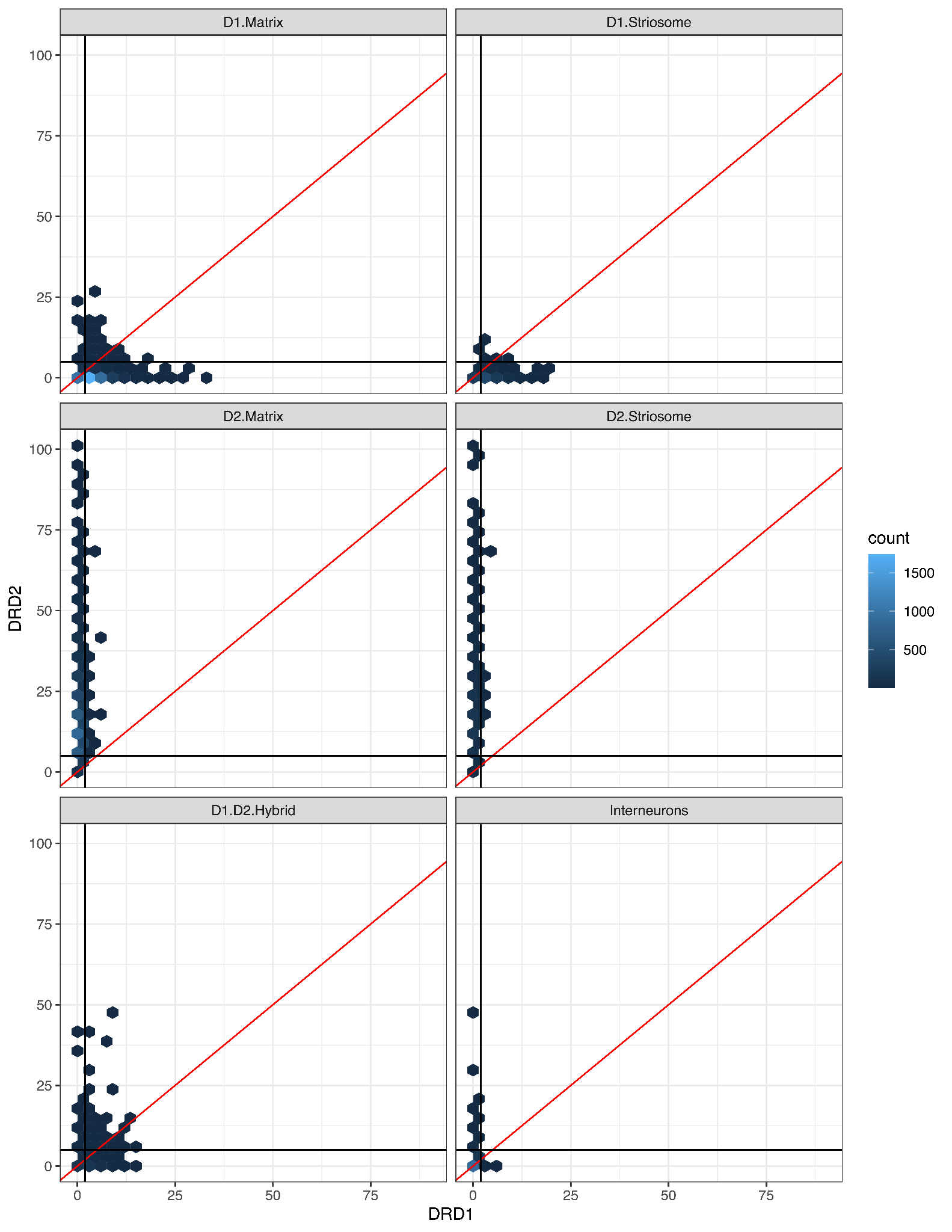


#### Supplementary Figure 3. Co-expression heatmap of single cells expressing *DRD1* and *DRD2* in major neuronal subpopulations.

#### Gene expression counts for *DRD1* on x-axis and *DRD2* on y-axis. Co-expression profiles indicates comparatively more consistent expression of both *DRD1* and *DRD2* in D1/D2-hybrid medium spiny neurons. Exact numbers of each cell type are reported in Source data Figure1_source_data.xlsx.

####
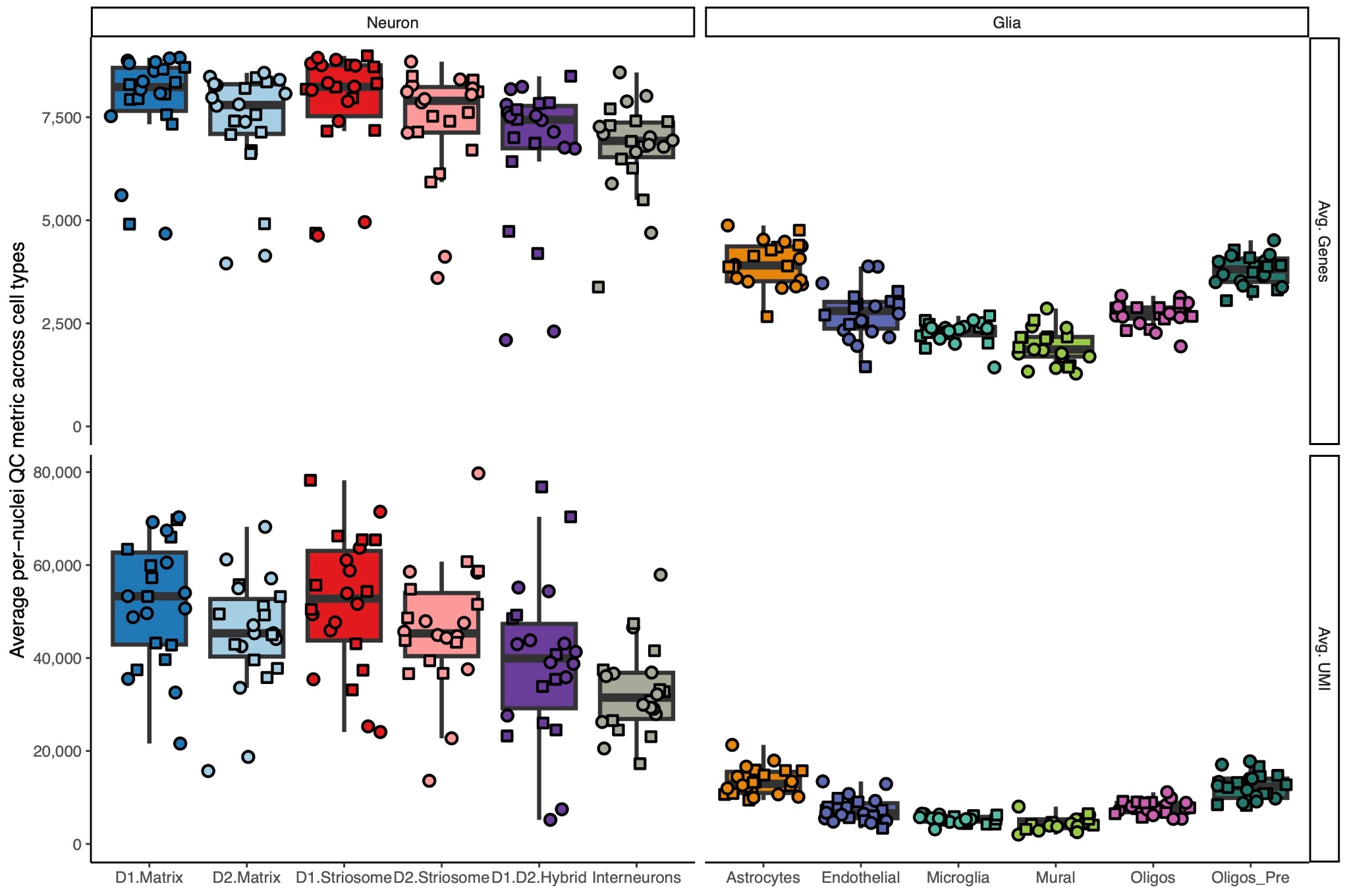
 Supplementary Figure 4. Cluster-specific quality control metrics of striatal cell types after QC filtering and annotation.

#### Quality control metrics, average number genes per cell and unique molecular identifiers (UMIs) per cell, show neuron and glia-specific differences in sample quality. Each point is a unique biospecimen. Round points represent female and squares represent male subjects. N = 22 biospecimens are from caudate and putamen are plot from M = 12 biologically independent individuals from one experiment.

####

####
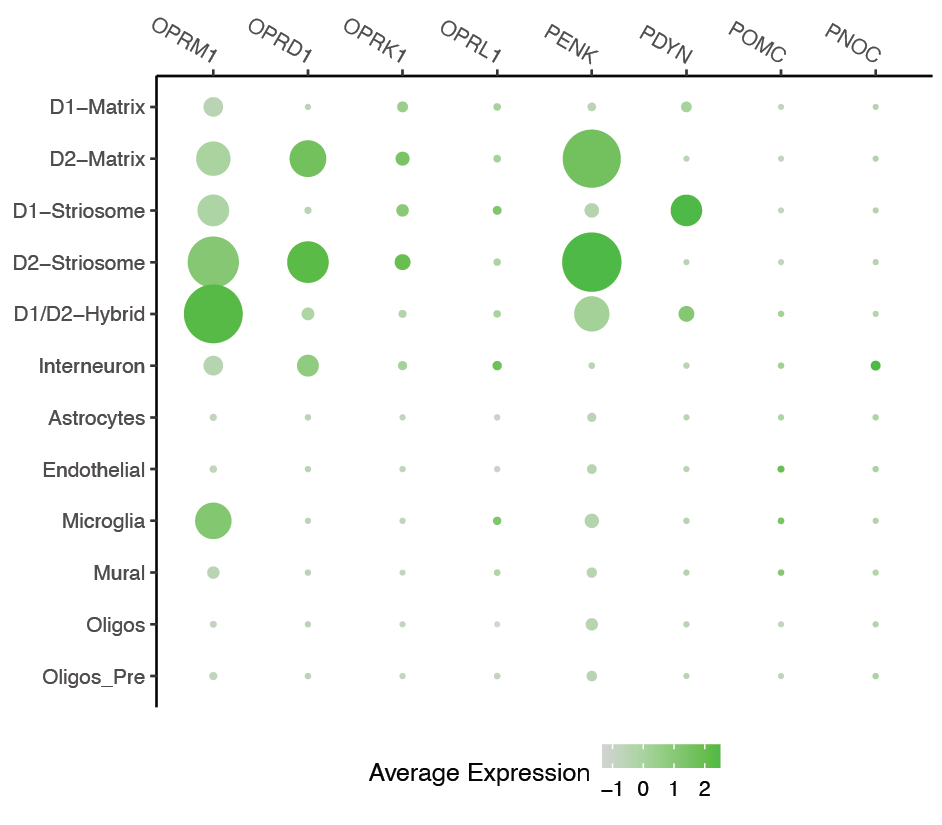


#### Supplementary Figure 5. Expression patterns of striatal opioid receptor and endogenous ligands

Dot plots of the opioid receptor and endogenous ligands across the annotated striatal cell types. The normalized expression patterns are averaged across all cells and subjects. Source data for this plot are in “Figure_supplement_source_data.xlsx” tab Fig_S5.


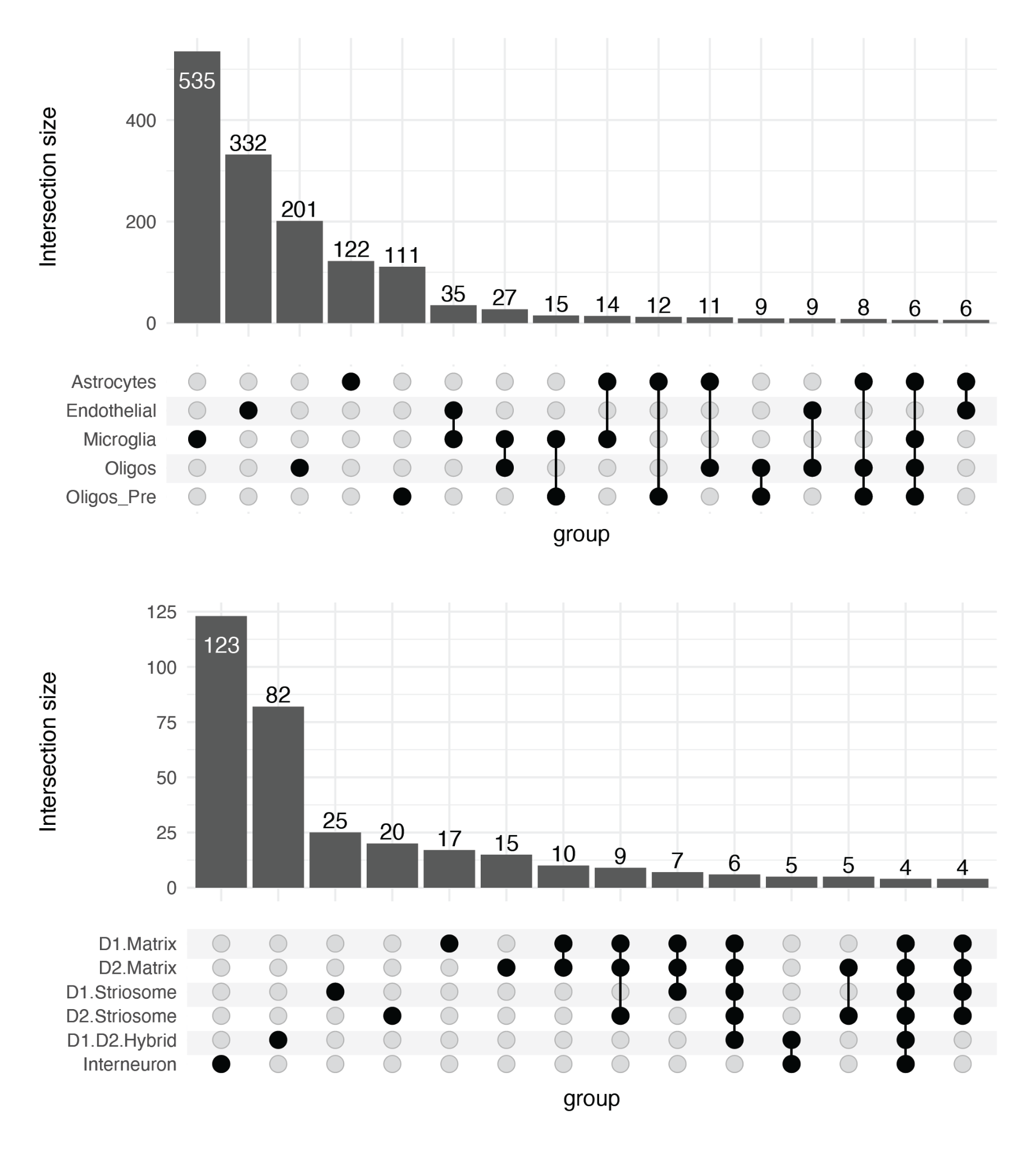


#### Supplementary Figure 6. Upset plot of overlapping differentially expressed genes in OUD

Upset plot showing the overlaps in significantly differentially expressed genes (FDR < 0.05) across glia (top) or neurons (bottom). The histogram shows how many genes are in each intersection of cell types that share DEGs.


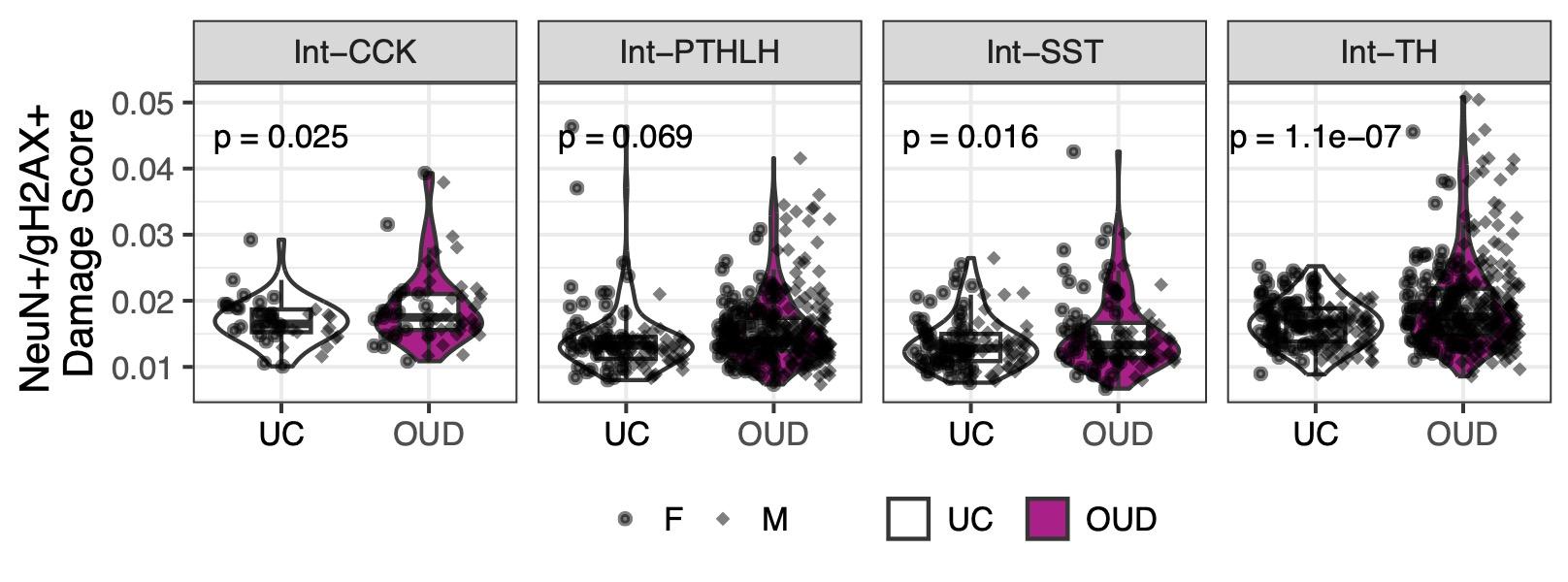


Supplementary Figure 7. Elevated DNA damage markers across interneuron subtypes associated with OUD.

Boxplot of cell-level neuronal DNA damage scores across striatal interneuron subtypes between unaffected and OUD subjects. One two-sided mixed effect linear regression test (1154 degrees of freedom) was used to compare the cell type interaction effect with the diagnosis of opioid use disorder (OUD) subjects on neuronal DNA damage score. Source data including the number of cells per cohort group are available in “Figure_supplement_source_data.xlsx” under Figure_S7.


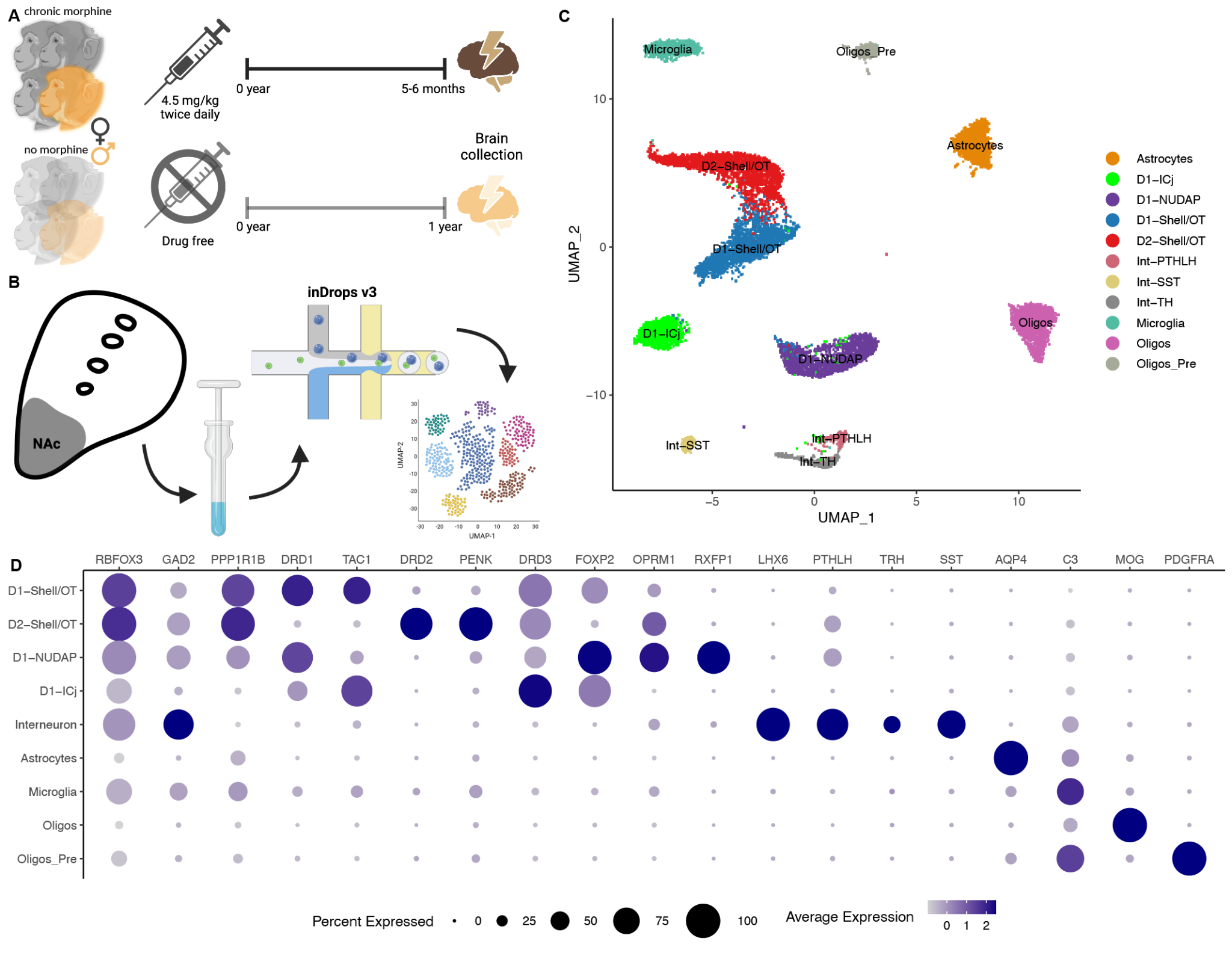


#### Supplementary Figure 8. Chronic morphine exposure and single nucleus RNA-seq of the rhesus macaque striatum

(A) Schematic of chronic morphine dosing strategy for 5-6 months prior to brain tissue collection in N=4 rhesus macaques to a total of 1,500 mg of total morphine exposure. N=4 control treatment subjects are matched by age, sex, and weight to the chronic morphine cohort and are unexposed to morphine or other drugs for 1 year prior to brain tissue collection. (B) Schematic of nucleus extraction from a tissue punch of the nucleus accumbens for inDrops single nuclei RNA-seq library synthesis and clustering. (C) UMAP projection of post-quality control and annotation of the rhesus macaque striatal cell types using a high-quality reference. (D) Dot plot showing the marker genes used to validate the annotations of the chronic morphine rhesus macaque striatal single nucleus RNA-seq dataset. Schematics in (A) created using BioRender.com. Source data for panels C and D including the number of cell types from N = 8 biologically independent individuals are available in “Figure_supplement_source_data.xlsx”.


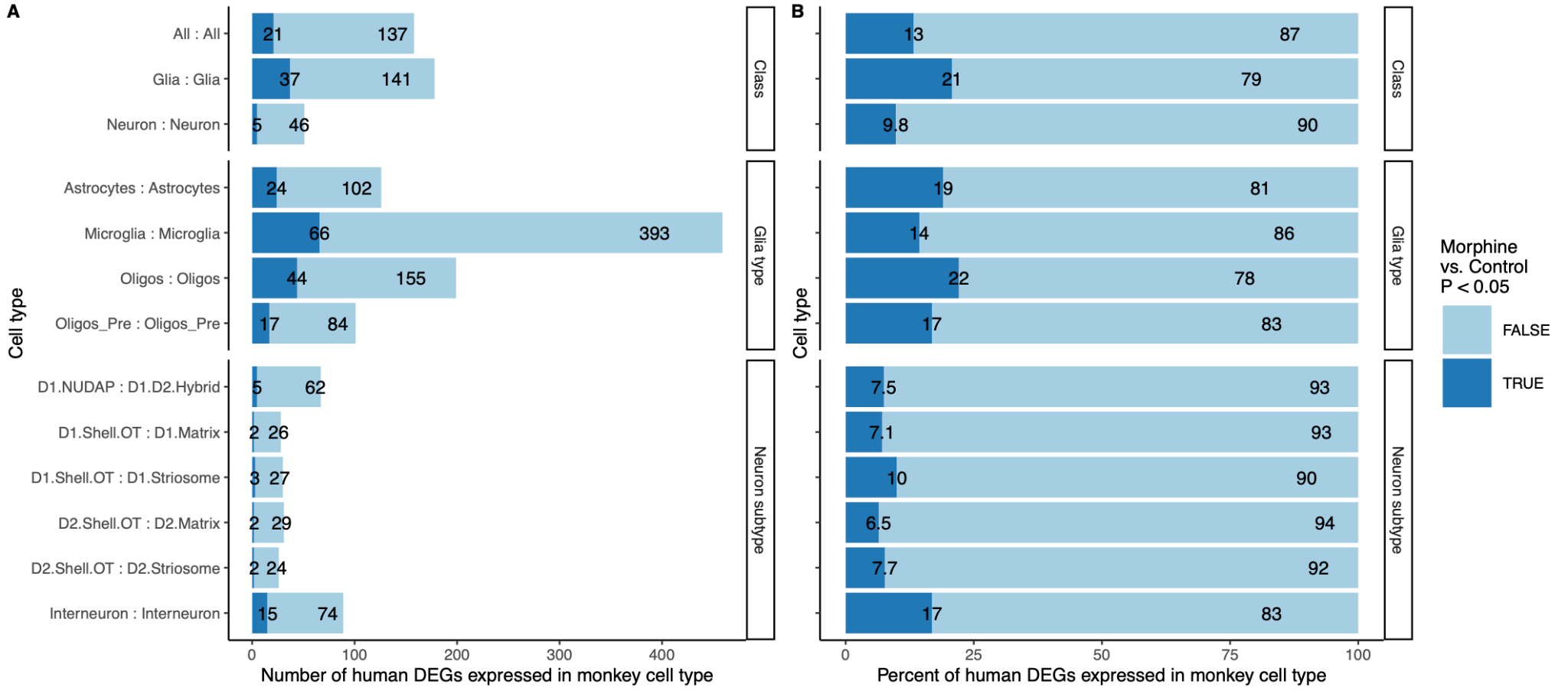


#### Supplementary Figure 9. Replication of human opioid use disorder differential expression in rhesus macaque striatal cell types

Barplots in the number (A) or percent (B) of human differentially expressed genes (DEGs) in opioid use disorder within dorsal striatal cell types that are also differentially expressed at replication P < 0.05 in the corresponding rhesus macaque ventral striatal cell types in chronic morphine exposure.

####
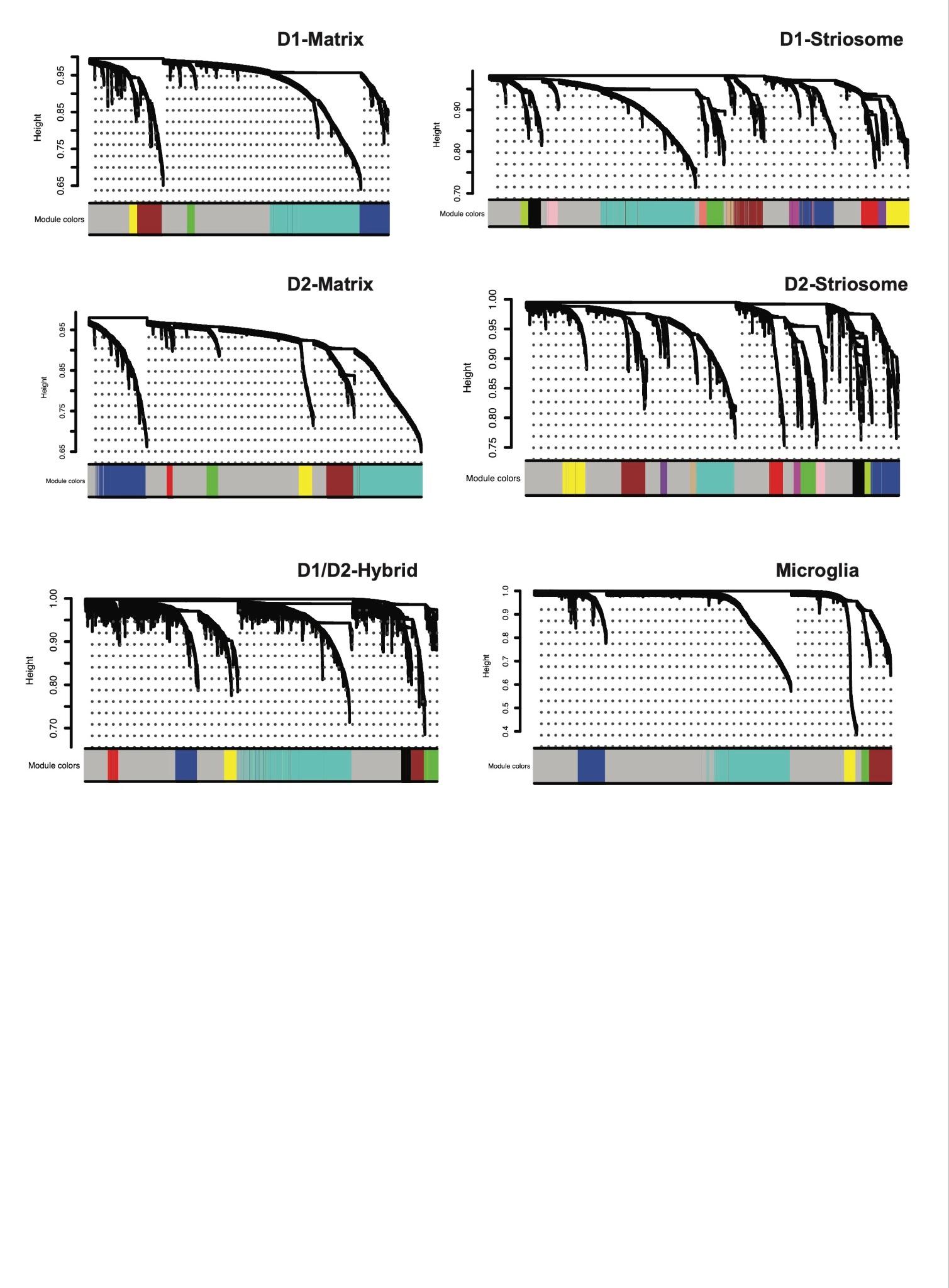
 Supplementary Figure 10. Dendrograms of gene co-expression networks among modules in medium spiny neuron cell types and microglia.

Dendrograms from WGCNA showing the average linkage hierarchical clustering of genes per cell type.


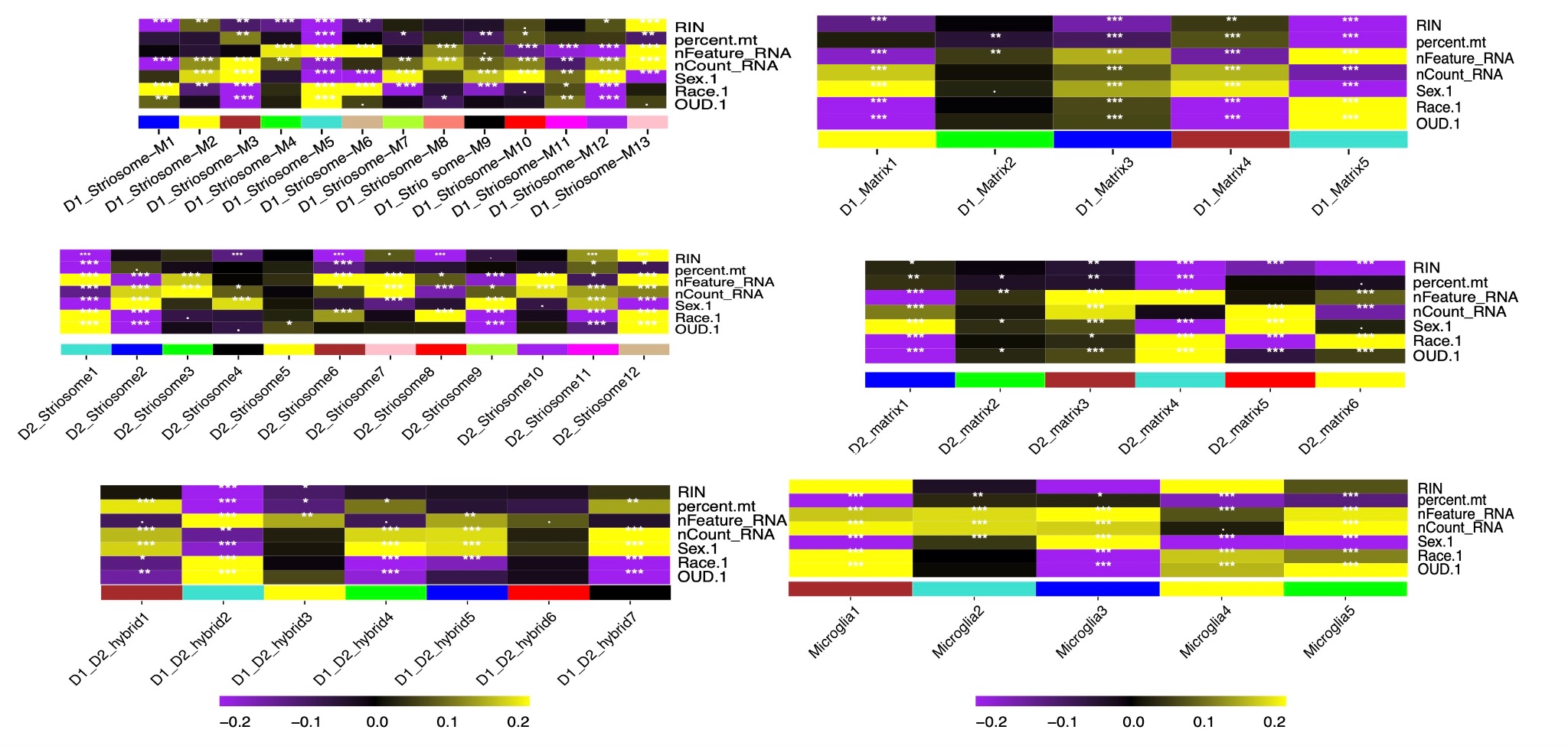


#### Supplementary Figure 11. Module-trait relationships for striatal cell types.

Correlation between modules (except grey module) and each of 6 traits (RIN, percent_mt, nFeature_RNA, nCount_RNA, Sex, Race and OUD). Correlation coefficient value was suggested by color (purple: negative correlation, yellow: positive correlation). ***: FDR < 0.001, **: FDR < 0.01, *: FDR < 0.05. Source data and exact FDR values are in Table S19.


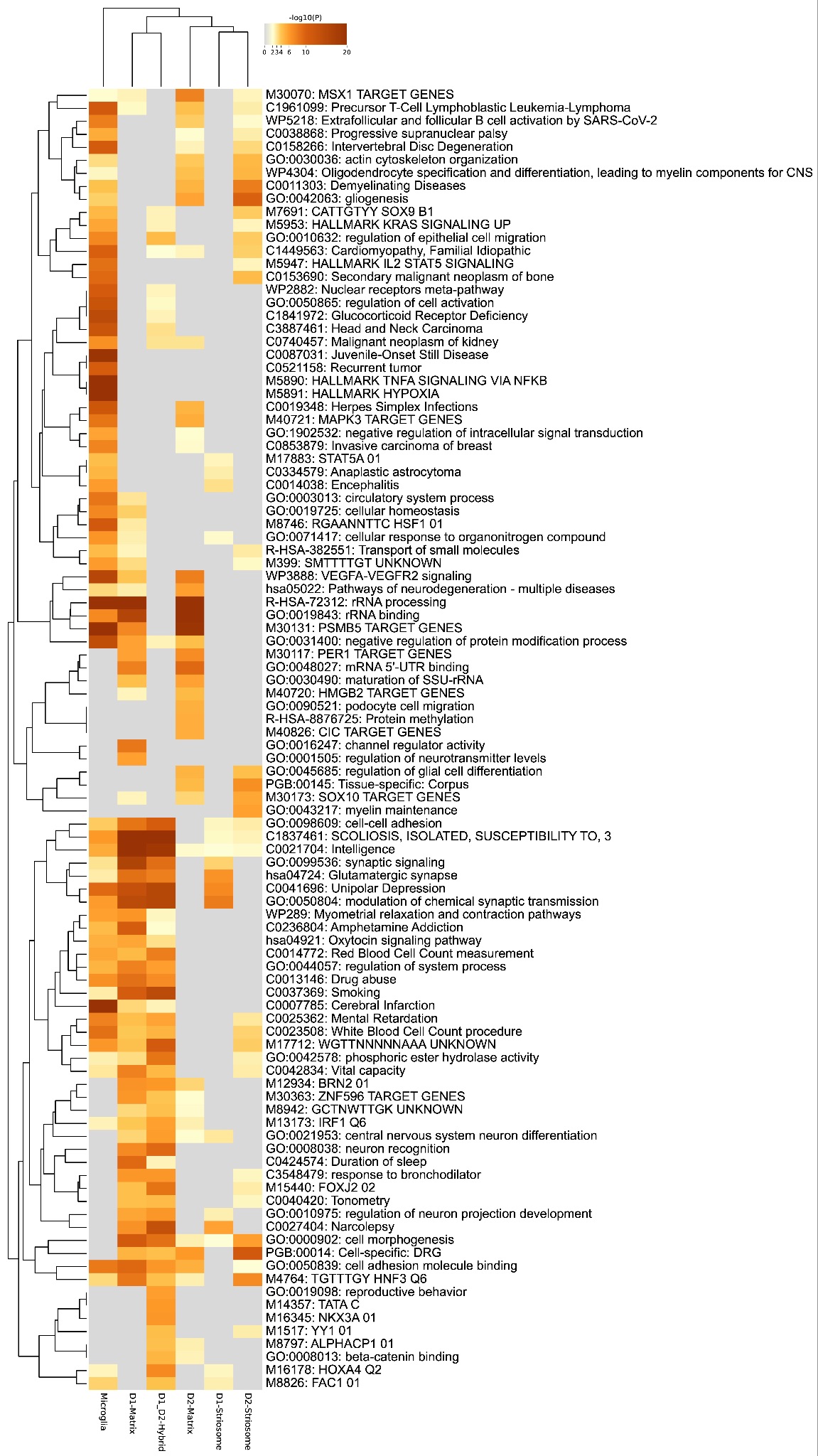


#### Supplementary Figure 12. Top 100 enriched pathways among genes within significant OUD-associated modules in medium spiny neuron subpopulations and microglia.

#### Source data are available in Supplemental Table S18.

####


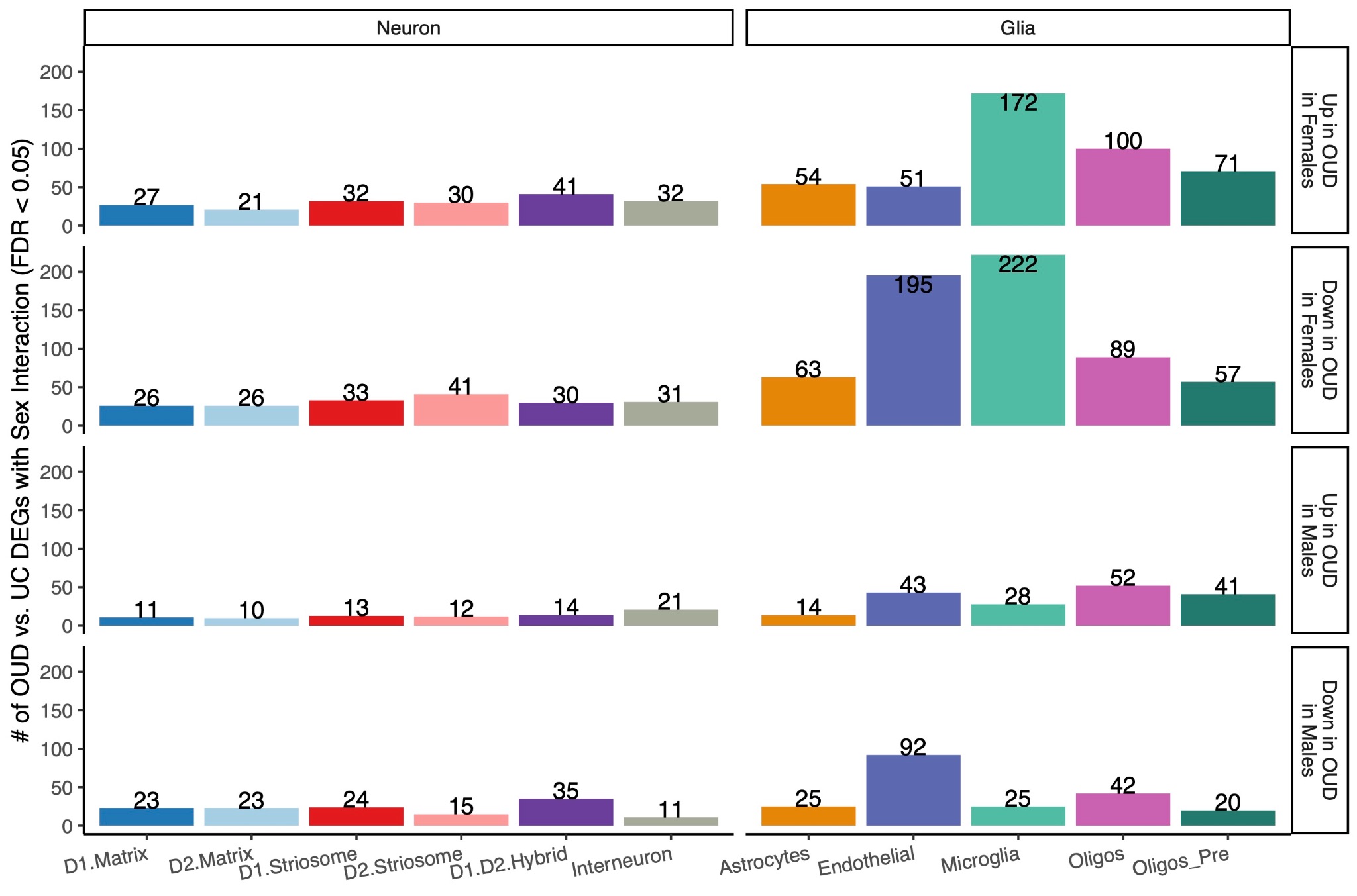


Supplementary Figure 13. The effect of biological sex and OUD diagnosis in striatal cell types.

Barplot of striatal cell types with significant interaction effects (FDR < 0.05) stratified by cell type and main effects. The genes are grouped into four groups by the direction of the interaction fold-change and whether the OUD effect is larger in females or males.


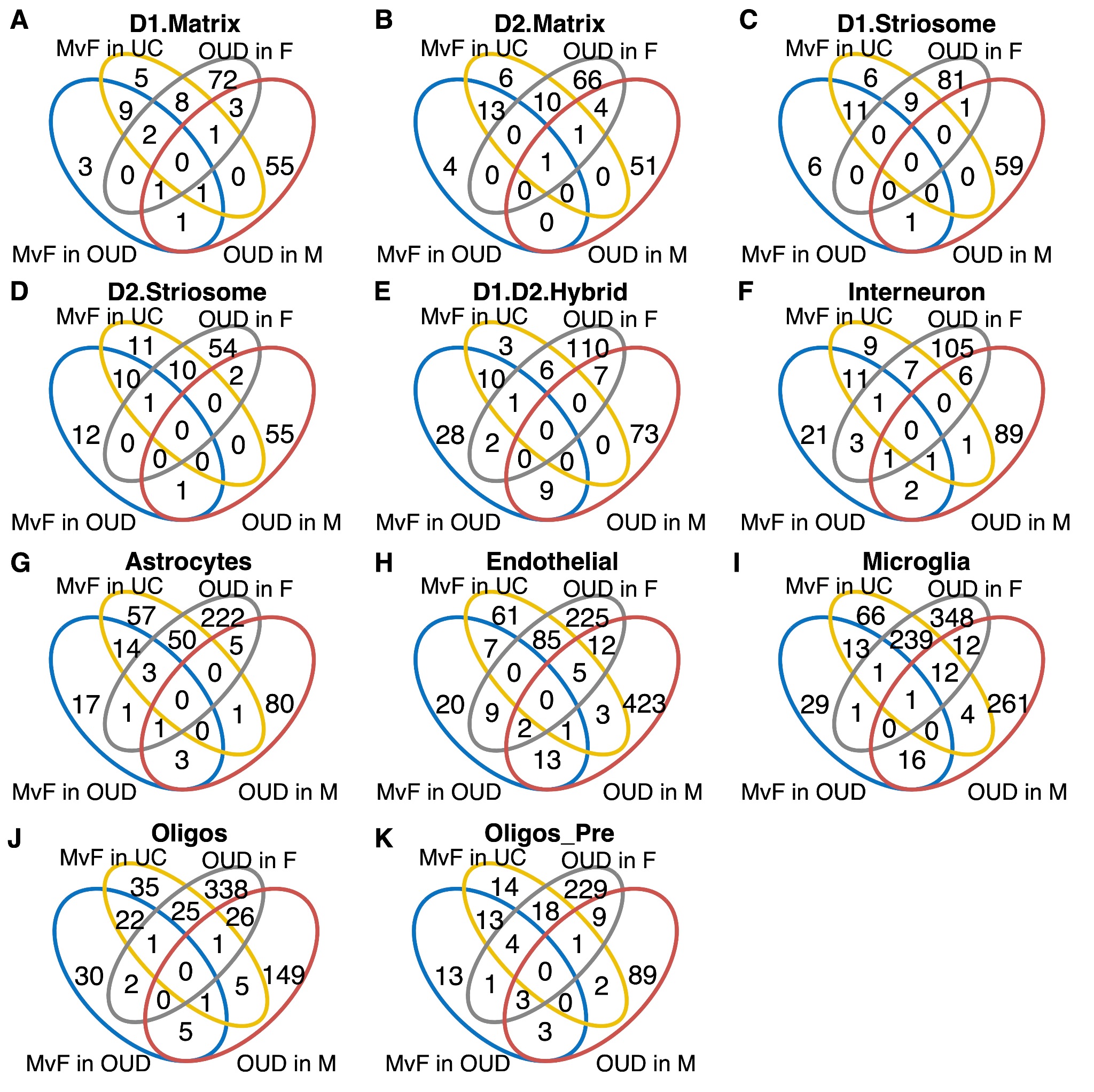


Supplementary Figure 14. Limited overlap of sex-specific changes in OUD compared to unaffected individuals with differences between sexes in OUD or unaffected individuals.

Four-way Venn diagram showing the number of overlapping differentially expressed genes in the following groups (FDR < 0.05) within striatal neuronal cell types (A-F) and glial cell types (G-K). MvF in UC (yellow oval): within the subset of unaffected individuals, the differentially expressed genes between females vs. males. MvF in OUD (blue oval): within the subset of OUD individuals, the differentially expressed genes between females vs. males (reference = male). OUD in F (gray oval): within the subset of female individuals, the differentially expressed genes between OUD vs. unaffected individuals. OUD in M (red oval): within the subset of male individuals, the differentially expressed genes between OUD vs. unaffected individuals.
