# Supplementary material for: Single nuclei transcriptomics in human and non-human primate striatum in opioid use disorder
Source: Nat Commun. 2024 Jan 31;15:878. doi: 10.1038/s41467-024-45165-7 (PMC10831093; doi:10.1038/s41467-024-45165-7)
Supplement: Supplementary file 4 — Description of Additional Supplementary Files [file 41467_2024_45165_MOESM4_ESM.docx]

### Description of Additional Supplementary Files

#### File Name: Supplementary Data 1-S1. Comparison of quality control metrics by OUD diagnosis

False discovery rate-corrected two-sided t-tests comparing aggregated single nuclei RNA-se quality control metrics between UC and OUD individuals across the caudate or putamen.

#### File Name: Supplementary Data 1-S2. Comparison of proportion of cell types by OUD diagnosis

False discovery rate-corrected multiple two-sided mixed effect linear regression test for any difference in cell type proportion between UC vs. OUD controlling for covariates as fixed effects and individual as a random effect.

#### File Name: Supplementary Data 1-S3. Significant marker genes of striatal cell types

Significant marker genes for each striatal cell type using the Seurat FindMarkers() function with default parameters to compute one-vs-all comparison using two-sided Wilcoxon-rank sum test. We computed the FDR across all cell types and significant marker genes are reported at FDR < 0.05.

#### File Name: Supplementary Data 1-S4. Significant marker TF-gene modules of striatal cell types

Significant marker TF-gene module activation for each striatal cell type using two-sided t-tests comparing the TF-gene module AUCell scores of each cell type with all others. The false discovery rate was computed across all cell types and significant marker genes are reported at FDR < 0.05.

#### File Name: Supplementary Data 1-S5. Postmortem human individual-level and tissue-level metadata

Description of the biospecimen-level and individual-level metadata describing relevant covariates and opioid use disorder diagnoses.

#### File Name: Supplementary Data 1-S6. Differentially expressed genes in UC vs. OUD within striatal cells

Outputs of two-sided limma-based linear regression test for differential expression across striatal cell type. The differential expression FDR was calculated using the swfdr method to increase power by modeling power with an independent factor, average expression. We calculated FDR within each cell type comparison (adj.P.Val.Within) and between all cell type comparisons (adj.P.Val.Between) and report the more conservative correction between cell types in the main text and figures, adj.P.Val.Between.

#### File Name: Supplementary Data 1-S7. Enriched pathways in UC vs. OUD, main comparison

Outputs of the gene-set pathway enrichment analyses from two-sided rank-based GSEA enrichment test using the main differential expression from UC vs. OUD. We calculated FDR across all cell type comparisons using swfdr modeling power by the number of genes in a gene set.

#### File Name: Supplementary Data 1-S8. Enriched TF-gene modules in UC vs. OUD

Outputs of differential pseudo-bulk average transcription factor-gene regulatory network activation in striatal cell types using two-sided linear regression test to control for covariates.

#### File Name: Supplementary Data 1-S9. Clustered neuronal pathways in UC vs. OUD, main comparison

Neuronal pathways from Table S7 with additional clustering to identify related, enrich pathways. The cluster number corresponds to those in Figure 2B-C. No additional statistical manipulations performed.

#### File Name: Supplementary Data 1-S10. Comparison of neuronal DNA damage score by OUD diagnosis

Outputs of differential pseudo-bulk average DNA damage score activation in striatal neurons using two-sided linear regression test to control for covariates, 15 degrees of freedom.

#### File Name: Supplementary Data 1-S11. Clustered glial pathways in UC vs. OUD, main comparison

Glial pathways from Table S7 with additional clustering to identify related, enrich pathways. The cluster number corresponds to those in Figure 4B-C. No additional statistical manipulations performed.

#### File Name: Supplementary Data 1-S12. Differentially expressed genes in UC vs. OUD subset to female or male

Similar outputs to Table S6 from two-sided limma-based linear regression tests for differential expression across striatal cell type. The differential expression FDR was calculated using the swfdr method to increase power by modeling power with an independent factor, average expression. These differential expression tables assess UC vs. OUD in female or male individuals (SexF and SexM, respectively).

#### File Name: Supplementary Data 1-S13. Enriched female-biased pathways in UC vs. OUD

Similar outputs to Table S7 showing gene-set pathways using two-sided rank-based GSEA enrichment tests on the differential expression from UC vs. OUD that were biased to female individuals with OUD. We calculated FDR across all cell type comparisons using swfdr modeling power by the number of genes in a gene set.

#### File Name: Supplementary Data 1-S14. Enriched male-biased pathways in UC vs. OUD

Similar outputs to Table S7 showing gene-set pathway using two-sided rank-based GSEA enrichment tests on the differential expression from UC vs. OUD that were biased to male individuals with OUD. We calculated FDR across all cell type comparisons using swfdr modeling power by the number of genes in a gene set.

#### File Name: Supplementary Data 1-S15. Enriched pathways with a sex interaction in UC vs. OUD

Similar outputs to Table S7 showing gene-set pathway using two-sided rank-based GSEA enrichment tests on the differential expression from UC vs. OUD that had a differential interaction score between DEGs in females vs. in male individuals. We calculated FDR across all cell type comparisons using swfdr modeling power by the number of genes in a gene set.

#### File Name: Supplementary Data 1-S16. Enriched pathways from Piechota et al. 2010

Gene-set enrichment analysis using two-sided rank-based GSEA enrichment tests with gene sets from Piechota et al. 2010, gene pathways after drug exposure in male mouse striatum, with differentially expressed genes. We calculated FDR across all cell type comparisons using swfdr modeling power by the number of genes in a gene set.

#### File Name: Supplementary Data 1-S17. Detected transcription factor (TF)-gene relationships

The transcription factor-gene regulatory relationships identified using the pySCENIC protocol to build gene regulatory networks. These TF-gene relationships were identified across multiple runs and across multiple individuals with and without OUD.

#### File Name: Supplementary Data 1-S18. Significantly enriched pathways from gene co-expression modules significantly correlated with OUD in medium spiny neuron subpopulations and microglia.

Pathway enrichment analysis on gene members of co-expression modules across cell types in human caudate and putamen. Pathways from co-expression modules were significantly correlated with medium spiny neurons and microglia cell types from individuals with OUD.

#### File Name: Supplementary Data 1-S19. Weighted gene co-expression analysis (WGCNA) across medium spiny neuron subpopulations and microglia.

Weighted gene co-expression network analysis (WGCNA) was used to generate gene co-expression modules, with the network structure generated on each major cell type separately.

File Name: Supplementary Data 1-20. Gene members of each module identified from WGCNA across medium spiny neuron subpopulations and microglia.

File Name: Supplementary Data 1-21. Trait-module correlations.

File Name: Supplementary Data 1-22. Trait-module correlations FDR values.

File Name: Supplementary Data 1-23. Gene members of modules used in GO pathway analyses.

File Name: Supplementary Data 1-24. GO pathway results for WGCNA modules.

File Name: Supplementary Data 1-25. Gene members in top 3 GO pathways.

File Name: Supplementary Data 1-S26. Postmortem rhesus macaque individual-level and tissue-level metadata

Description of the individual-level metadata describing relevant covariates and chronic morphine dosing schedules and prior drug exposure.
