# Supplementary material for: Single nuclei transcriptomics in human and non-human primate striatum in opioid use disorder
Source: Nat Commun. 2024 Jan 31;15:878. doi: 10.1038/s41467-024-45165-7 (PMC10831093; doi:10.1038/s41467-024-45165-7)
Supplement: Supplementary file 5 — Reporting Summary [file 41467_2024_45165_MOESM5_ESM.pdf]

nature portfolio

Corresponding author(s): Ryan W. Logan and Andreas R. Pfenning

Last updated by author(s): Dec 29, 2023

Reporting Summary

Nature Portfolio wishes to improve the reproducibility of the work that we publish. This form provides structure for consistency and transparency in reporting. For further information on Nature Portfolio policies, see our Editorial Policies and the Editorial Policy Checklist.

Statistics

For all statistical analyses, confirm that the following items are present in the figure legend, table legend, main text, or Methods section.

n/a	Confirmed	
☐	☒	The exact sample size (*n*) for each experimental group/condition, given as a discrete number and unit of measurement
☐	☒	A statement on whether measurements were taken from distinct samples or whether the same sample was measured repeatedly
☐	☒	The statistical test(s) used AND whether they are one- or two-sided<br>*Only common tests should be described solely by name; describe more complex techniques in the Methods section.*
☐	☒	A description of all covariates tested
☐	☒	A description of any assumptions or corrections, such as tests of normality and adjustment for multiple comparisons
☐	☒	A full description of the statistical parameters including central tendency (e.g. means) or other basic estimates (e.g. regression coefficient) AND variation (e.g. standard deviation) or associated estimates of uncertainty (e.g. confidence intervals)
☐	☒	For null hypothesis testing, the test statistic (e.g. *F*, *t*, *r*) with confidence intervals, effect sizes, degrees of freedom and *P* value noted<br>*Give P values as exact values whenever suitable.*
☒	☐	For Bayesian analysis, information on the choice of priors and Markov chain Monte Carlo settings
☐	☒	For hierarchical and complex designs, identification of the appropriate level for tests and full reporting of outcomes
☐	☒	Estimates of effect sizes (e.g. Cohen's *d*, Pearson's *r*), indicating how they were calculated

Our web collection on statistics for biologists contains articles on many of the points above.

Software and code

Policy information about availability of computer code

Data collection	10X Genomics Platform for Single Nuclei RNA-sequencing of human postmortem caudate and putamen; InDrop v3 Platform for Single Nuclei RNA-sequencing of striatum from rhesus macaques
Data analysis	Single nuclei RNA-seq data processing and downstream analyses of human dorsal striatum are collected in the github repository https://github.com/pfenninglab/Logan_Striatum_snRNA-seq. Single nuclei RNA-seq processing and downstream analyses of the rhesus macaque striatum are collected in the github repository https://github.com/pfenninglab/M clean_chronic_opioid_monkey_snRNA-seq.<br><br>STARSolo v2.7.9a count using human genome GRCh38.p13 and rhesus macaque rheMac10<br>Alternate cell annotations in rhesus macaque using liftoff tool v1.6.3<br>SoupX v1.3.0 for ambient RNA contamination<br>DropletQC<br>Doublets with scds<br>Damaged nuclei with miQC<br>Seurat v4 for cell clustering with SCTransform<br>glmGamPoi v3.18<br>limma-voom v3.46<br>pySCENIC v0.12.1<br>hdWGCNA v0.2.26

For manuscripts utilizing custom algorithms or software that are central to the research but not yet described in published literature, software must be made available to editors and reviewers. We strongly encourage code deposition in a community repository (e.g. GitHub). See the Nature Portfolio guidelines for submitting code & software for further information.

Data

Policy information about availability of data

All manuscripts must include a data availability statement. This statement should provide the following information, where applicable:

- Accession codes, unique identifiers, or web links for publicly available datasets
- A description of any restrictions on data availability
- For clinical datasets or third party data, please ensure that the statement adheres to our policy

Single nuclei RNA-seq data processing and downstream analyses of human dorsal striatum are collected in the github repository https://github.com/pfenninglab/Logan_Striatum_snRNA-seq. Single nuclei RNA-seq processing and downstream analyses of the rhesus macaque striatum are collected in the github repository https://github.com/pfenninglab/M clean_chronic_opioid_monkey_snRNA-seq. The raw sequencing reads and annotated Seurat objects for both human and rhesus macaque studies are uploaded to GEO under SuperSeries accession number GSE233279. A browsable webportal of the human dorsal striatum single nuclei transcriptomes are on the CZ CellxGene Discovery webportal (https://cellxgene.cziscience.com/collections/cec4ef8e-1e70-49a2-ae43-1e6bf1fd5978).

Research involving human participants, their data, or biological material

Policy information about studies with human participants or human data. See also policy information about sex, gender (identity/presentation), and sexual orientation and race, ethnicity and racism.

Reporting on sex and gender	Gender was not collected from self-report as part of human cohort. Biological sex was recorded for the human cohort. Differential expression gene analysis between unaffected individuals and individuals with OUD were calculated as the average between sexes. We also performed analyses restricted to each sex and reported sex-biased gene expression signatures in unaffected and OUD cohorts.
Reporting on race, ethnicity, or other socially relevant groupings	Race and ethnicity are reported in the metadata within the supplement. Race and/or ethnicity were excluded as a covariate in the analyses.
Population characteristics	Unaffected individuals and individuals with opioid use disorder were collected from the University of Pittsburgh Brain Bank and local to Allegheny County. Cause of death, along with any medical/clinical diagnoses were confirmed via rigorous record reviews by a team of psychologists, psychiatrists, and physicians. Extensive next-of-kin interviews were also conducted. Age was considered as a covariate in our analyses, reported in the metadata within the supplement. Co-occurring medical and/or psychiatric disorders were excluded as covariates from the analyses.
Recruitment	Postmortem human brain samples were obtained, following consent from the next of kin, during autopsies conducted by the Allegheny County Office of the Medical Examiner (Pittsburgh, PA). Consent was obtained from next-of-kin and procedures were approved by the University of Pittsburgh's committee for Oversight of Research and Clinical Training Involving Decedents and Institutional Review Board for Biomedical Research. An independent committee of clinicians made consensus, lifetime DSM-IV diagnoses for each individual using the results of an expanded psychological autopsy, including structured interviews with family members and review of medical records, as well as toxicological and neuropathological reports. The same approach was used to confirm the absence of lifetime psychiatric and neurologic disorders in the unaffected comparison subjects. All procedures were approved by the University of Pittsburgh Committee for Oversight of Research and Clinical Training Involving Decedents and Institutional Review Board for Biomedical Research. Each individual meeting diagnostic criteria for opioid use disorder at the time of death (n = 6) was matched with an unaffected comparison subject (n = 6) for sex and as closely as possible for age and PMI (see Table S5). The duration of illness for each OUD subject was at least four years prior to death.
Ethics oversight	Consent was obtained from next-of-kin and procedures were approved by the University of Pittsburgh's committee for Oversight of Research and Clinical Training Involving Decedents and Institutional Review Board for Biomedical Research.

Note that full information on the approval of the study protocol must also be provided in the manuscript.

Field-specific reporting

Please select the one below that is the best fit for your research. If you are not sure, read the appropriate sections before making your selection.

☒ Life sciences ☐ Behavioural & social sciences ☐ Ecological, evolutionary & environmental sciences

For a reference copy of the document with all sections, see nature.com/documents/nr-reporting-summary-flat.pdf

Life sciences study design

All studies must disclose on these points even when the disclosure is negative.

Sample size	No a priori sample size alculations were performed. Instead, we assembled a cohort that was well matched between unaffected individuals and individuals with opioid use disorder by sex, age, postmortem interval, and RNA integrity number. The overall cohort is balanced for sex in both unaffected and opioid use disorder groups. We optimized the single nuclei experiment to capture more nuclei per biospecimen (N=24 biospecimens) given the disparate differences between the most and least prevalent cell types (60% oligodendrocytes vs. <1% interneuron subtypes) in postmortem human striatal tissues. We collected ~140,000 nuclei post sequencing and ~98,ooo post quality control resulting in

	~100 nuclei of the rarest population, interneurons, post QC per individual to have sufficient sampling of genes per cell type for differential analyses. We also deeply sequenced each cell beyond the average human or mouse single cell atlas studies and in line with published theoretical calculations near the recommendation of 1 read per cell per gene based on ref PMID: 32034137 than previous studies. This resulted in an average of 13,000 UM Is per cell capturing nearly 20,000 expressed genes across all cell types and biospecimens.
Data exclusions	We excluded samples where QC metrics revealed failure to capture individuals cells (N=2). Both of these samples were from the caudate of unaffected individuals. The putamen from these same subjects was included in the analyses.
Replication	Single nuclei RNA-sequencing experiments in human postmortem brains were conducted in group-balanced batches for both caudate and putamen from the same individual. Caudate and putamen were compared to each other for replication within individual, then integrated for subsequent analyses. Rhesus macaque single nuclei RNA-sequencing experiments were conducted with N=1 for each striatum from each individual. Findings from human striatum were directly compared to rhesus macaque datasets (PMID: 34727523), yielding high convergence of annotated and clustered cell types. Human and rhesus macaque findings were also directly compared to rodent studies of substance use (PMID: 20459597) and rodent studies of DNA damage (PMID: 36170369), both of which yielded high convergence, subsequently discussed in the main results.
Randomization	Postmortem human and rhesus macaque cohorts were not randomized. Cohorts were selected to have matching covariates relevant to each cohort. The human cohort was matched for sex, age, postmortem interval, and RNA integrity number. These were covariates in statistical analyses except for sex where it was contrasted to find the average effect between sexes. The rhesus macaque cohort was matched for age, sex, and weight, and a matching variable was used to describe the 1-1 matching covariate between morphine and control subjects.
Blinding	Blinding was not possible at time of sample collection. To minimize potential confounds, samples were collected and processed in batches, balanced across diagnosis or experimental groups. Human postmortem tissues were processed in 6 batches, 4biospecimens at a time 2 from each diagnosis group. Rhesus macaque postmortem tissues were collected in 2 batches, 4 biospecimens at a time, 2 from each treatment group. Blinding is not possible at the time of data analyses and all software used to analyze the data are standard practice in genomics.

Reporting for specific materials, systems and methods

We require information from authors about some types of materials, experimental systems and methods used in many studies. Here, indicate whether each material, system or method listed is relevant to your study. If you are not sure if a list item applies to your research, read the appropriate section before selecting a response.

Materials & experimental systems

n/a	Involved in the study	
☒	☐	Antibodies
☒	☐	Eukaryotic cell lines
☒	☐	Palaeontology and archaeology
☐	☒	Animals and other organisms
☒	☐	Clinical data
☒	☐	Dual use research of concern
☒	☐	Plants

Methods

n/a	Involved in the study	
☒	☐	ChIP-seq
☒	☐	Flow cytometry
☒	☐	MRI-based neuroimaging

Animals and other research organisms

Policy information about studies involving animals; ARRIVE guidelines recommended for reporting animal research, and Sex and Gender in Research

Laboratory animals	Eight adult rhesus monkeys (macaca mulatta) weighing between 6.0 and 13.0 kg served as subjects in the present study. Four subjects (mean age: 12 years, range: 9-18 years) received daily morphine treatment for 5 months (see below); four additional subjects, matched for age, weight, and sex, served as experimental controls (mean age: 14 years, range: 11-21 years).
Wild animals	N/A
Reporting on sex	Four subjects (3 male, 1 female) received daily morphine treatment for 5 months (see below); four additional subjects, matched for age, weight, and sex, served as experimental controls (3 male, 1 female).
Field-collected samples	This study did not involve samples collected in the field.
Ethics oversight	The rhesus macaque study were performed at McLean Hospital, a facility that is licensed by the U.S. Department of Agriculture, and all rhesus macaque experimental protocols were approved by the Institutional Animal Care and Use Committee at McLean Hospital.

Note that full information on the approval of the study protocol must also be provided in the manuscript.

Plants

Seed stocks	*Report on the source of all seed stocks or other plant material used. If applicable, state the seed stock centre and catalogue number. If plant specimens were collected from the field, describe the collection location, date and sampling procedures.*
Novel plant genotypes	*Describe the methods by which all novel plant genotypes were produced. This includes those generated by transgenic approaches, gene editing, chemical/radiation-based mutagenesis and hybridization. For transgenic lines, describe the transformation method, the number of independent lines analyzed and the generation upon which experiments were performed. For gene-edited lines, describe the editor used, the endogenous sequence targeted for editing, the targeting guide RNA sequence (if applicable) and how the editor was applied.*
Authentication	*Describe any authentication procedures for each seed stock used or novel genotype generated. Describe any experiments used to assess the effect of a mutation and, where applicable, how potential secondary effects (e.g. second site T-DNA insertions, mosiacism, off-target gene editing) were examined.*
